# Adolescents’ Psychoactive Substance Use During the First COVID-19 Lockdown: A Cross Sectional Study in Italy

**DOI:** 10.1007/s10566-022-09701-0

**Published:** 2022-07-27

**Authors:** Silvia Biagioni, Federica Baldini, Marina Baroni, Sonia Cerrai, Francesca Melis, Roberta Potente, Marco Scalese, Sabrina Molinaro

**Affiliations:** 1 Institute of Clinical Physiology, National Research Council of Italy, Via Moruzzi 1, 56124 Pisa, Italy; 2 Department of Surgical, Medical and Molecular Pathology and Critical Care Medicine, University of Pisa, Pisa, Italy; 3 Department of Social Sciences and Economics, Sapienza University of Rome, Rome, Italy

**Keywords:** Adolescents, Psychoactive substance use, Covid-19, Lockdown, Risk behaviours

## Abstract

**Background:**

Italy was one of the first European countries to be affected by Covid-19. Due to the severity of the pandemic, the Italian government imposed a nationwide lockdown which had a great impact on the population, especially adolescents. Distance-learning, moving restrictions and pandemic-related concerns, resulted in a particularly stressful situation.

**Objective:**

This cross-sectional study aims to analyse substance consumption and its associated factors during the Covid-19 lockdown imposed by the Italian government.

**Methods:**

ESPAD is a questionnaire that is administered yearly in Italian high schools. In 2020, it was administered online during dedicated hours of distance learning, collecting data from 6027 Italian students (52.4% were male) aged 15–19. Data collected from the 2020 questionnaire was matched with that collected in 2019, in order to make them comparable.

**Results:**

The prevalence of consumption of each substance decreased during the restriction period, and the most used substance during the lockdown period was alcohol (43.1%). There were some changes in factors associated with psychoactive substance use, especially painkillers and non-prescription drugs. For instance, unlike what was observed in the 2019 model, in 2020 spending money without parental control was associated with painkillers and non-prescription drug use while risk perception was not.

**Conclusions:**

The restrictions and the increased difficulties in obtaining psychoactive substances did not prevent their consumption, and students with particular risk factors continued to use them, possibly changing the substance type of substance. This information is useful in order to better understand adolescents’ substance use during the ongoing pandemic.

## Introduction

At the end of 2019, a new coronavirus disease namely Sars-CoV-2 broke out in China and it quickly affected many countries becoming a pandemic (ICTV-CSG, 2020). Italy was one of the first European countries to be affected and it registered the first confirmed case of COVID-19 infection in February 2020. In response to the growth of the pandemic, in March the government imposed a nationwide lockdown which implied social distancing and the restriction of movements except for necessity (e.g., work and health circumstances). In accordance with that, the lockdown demanded a change in people’s habits and this situation entailed negative consequences not only on the economic and social fabric of the country but also on the psychological wellbeing of the population (Caffo et al., 2020; Feregert et al., 2020; Indolfi & Spaccarotella, 2020).

Regarding adolescents, they had to face school closures, distance-learning, and home confinement, as well as pandemic-related concerns, thus resulting in a very stressful situation (Fegert et al., 2020; Guessoum et al., 2020). Related to this last assumption, several authors observed an increment of negative emotions such as boredom, irritability, nervousness, feelings of loneliness and worry among adolescents during lockdown (Orgilés et al., 2020).

Adolescence can be defined as a critical and vulnerable period (Casey et al., 2011; Larsene & Luna, 2018; Sturman & Moghaddam, 2011) during which it could be common to take part in risky behaviours (Hurrelmann, & Richter, 2006), like the use of legal (e. g. tobacco, alcohol, energy drinks) or illegal psychoactive substances. Concerning the Italian context, the 2019 ESPAD report observed that 32% of students smoked cigarettes in the last month and 19% of them reported daily use of cigarettes. The European estimates are respectively 20% and 10% (ESPAD Group, 2020). Moreover, in regards to other potential addictive behaviours, 59% of Italian adolescents reported using alcohol during the last 30 days, 27% used cannabis in their life, while other illegal substances (e.g. cocaine, heroin, hallucinogens, and ecstasy) had a lifetime prevalence of around 1–3% (ESPAD Group, 2020).

As postulated by the literature, several social and psychological factors may contribute to the use of both legal and illegal psychoactive substances among adolescents, for instance: family environment and functioning, peers influence, self-dissatisfaction, academic performance, and risk perception (Gray & Squeglia, 2018; Lee et al., 2018; Mathew et al., 2018; Salloum et al., 2018; Russell et al., 2020; Gravenstein et al., 2020; Henneberger et al., 2021; Kinnunen et al., 2022). These variables are also significant from a preventive point of view and are the main target of many prevention programs targeting adolescents (Ferri et al., 2013; EMCDDA, 2019; Xchange prevention registry). More in detail, literature findings pointed out that family could have an influential role on the use of psychoactive substances in terms of family support and quality of relationships, socio-economic status, parental monitoring, and parents psychoactive substances intake (Mathewet al., 2018). Moreover, a recent systematic review highlighted a potential influence of peers on the use of substances during adolescence in terms of two interconnected factors namely, peer selection and socialisation. Specifically, authors suggested that choosing friends who use psychoactive substances could be associated with social opportunities linked with this kind of risky behaviour (Henneberger et al., 2021). In regard to self-dissatisfaction, this factor could potentially contribute to the consumption of psychoactive substances considering that, in their longitudinal study, Lee et al. (2018) found a predictive role of self-esteem in the use of cannabis, cocaine, and binge drinking. Concerning the association between academic performance and psychoactive substance consumption, several authors observed a link between lower levels of academic achievement and the use of tobacco, alcohol, and cannabis among adolescents (Kinnunen et al., 2022). Lastly, as reported by literature, also risk perception constructs seem to have a role on both legal and illegal psychoactive substances use among adolescents (Salloum et al., 2018; Russell et al., 2020; Gravenstein et al., 2020).

Due to the particular condition related to the lockdown, some substances became very difficult to obtain and, in general, the consumption has dropped (EMCDDA, 2020). The lockdown made it more difficult to access illegal drugs markets and the forced cohabitation with parents involved, in some cases, a reduction in the use of tobacco and/or alcohol among adolescents. On the other hand, stressful life events, as the global pandemic of COVID-19 was, could increase addictive behaviours (Dubey et al., 2020) as well as the experience of negative emotions, especially boredom (Biolcati et al., 2018; Wegner & Flisher, 2009).

Analysing the use of substances and associated factors during the lockdown occurrence could contribute to understanding adolescents’ behaviours during stressful life events. Furthermore, it could provide directions for the preventive interventions in order to promote a better adaptation to the still ongoing pandemic and to prevent long-term difficulties among the adolescent population (Efuribe et al., 2020; Fegert et al., 2020; Guessoum et al., 2020).

Based on these assumptions, the aims of the present study were a) to describe the patterns of substance use under a significant stressful life event and b) to identify which of the associated variables impact substance use under these conditions, in comparison with data from ESPAD Italia 2019 and under the assumption that the expected changes without lockdown would have been marginal.

## Materials and Method

This study is based on data collected through ESPAD#iorestoacasa2020, an anonymous self-administered web questionnaire addressed to Italian high-school students. The questionnaire contains items on the patterns of use of psychoactive substances and additional questions on the topic of school, relationships with parents and friends, and family socioeconomic status.

Study methodology, school recruitment, and questionnaire administration were based on the ESPAD standardised protocol, in full compliance with anonymity and privacy, as reported elsewhere (Hibell et al., 2012). The Italian ESPAD®Italia study received an ethical review (n. 0,027,159/2019) by the Research Ethics and Bioethics Committee of the National Council of Research (CNR). Schools were initially selected by the ESPAD®Italia multistage stratified sampling procedure that ensures national representativeness, nonetheless due to COVID-19 it was not possible to reach all the sampled schools. For this reason, all the selected schools were asked to voluntarily participate, and 31% (78 schools) of them agreed. Data collection took place between April and the first half of May 2020. The questionnaire was administered online and the students responded from their homes during an hour of remote teaching. Despite the restrictions, the methodological adaptations (web-based questionnaire and distant learning setting for administration) have made it possible to obtain a large number of respondents, although, given these changes the data collected with ESPAD#iorestoacasa2020 cannot be considered completely representative of the Italian high schools’ student population and they are not directly comparable with ESPAD®Italia data collected in previous waves. For this reason, 2020 data were paired in terms of sex, age and macro-area of origin, with a sub-sample of students participating in the 2019 ESPAD®Italia wave.

The sample of the ESPAD#iorestoacasa2020 was composed of N = 6,027 Italian high-school students aged 15–19 years (male students = 52.4%; mean age 16.9 years; standard deviation 1.4) (Table 1). The paired 2019 sub-sample was composed by 6,027 students aged between 15 and 19 years (male students = 53.3%; mean age 17.0 years; standard deviation 1.4).

**Table 1:** General characteristic of the sample (Gender, age, and area of residence)

		Male (%)	Female (%)	Total (%)
*2020*				
Area of residence	Urban (City)	28.3	29.3	28.8
	Semi-Urban (Suburbs)	17.2	14.0	15.7
	Semi-Rural (Village)	44.6	45.4	45.0
	Rural (Countryside)	9.9	11.3	10.6
Age	15	21.9	20.6	21.3
	16	21.2	20.5	20.9
	17	18.7	20.3	19.5
	18	18.8	18.6	18.7
	19	19.4	20.0	19.7
*2019*				
Area of residence	Urban (City)	31.9	31.5	31.7
	Semi-Urban (Suburbs)	14.1	16.0	15.0
	Semi-Rural (Village)	43.2	43.8	43.5
	Rural (Countryside)	10.8	8.7	9.8
Age	15	19.4	21.8	20.5
	16	20.5	21.1	20.8
	17	19.7	19.6	19.7
	18	18.3	19.0	18.6
	19	22.0	18.6	20.4

The authors declare that they have no affiliations with or involvement in any organization or entity with any financial interest, or non-financial interest in the subject matter or materials discussed in this manuscript.

### Measures

ESPAD#iorestoacasa2020 and ESPAD®Italia 2019 collected information about substance use during the last 12 months and in the last 30 days. In 2020, the last 30 days overlap with the lockdown period. Alcohol, tobacco, e-cigarettes, energy drinks, painkillers without medical prescription, drugs without medical prescription, cannabis, cocaine, heroin, hallucinogens, and stimulants were considered. Information about the use of each substance was collected in terms of frequency (“1–2 times”, “3–9 times”, “10–19 times”, “20 times or more”) and then dichotomized as 0 = “not used” and 1 = “used” in the regression analyses. Due to the low prevalence of cocaine, heroin, hallucinogens, and stimulants use during the lockdown, these substances were grouped as “any other illegal substance” and it was not possible to provide information about frequency of use.

In regard to tobacco smoking, we also asked students if they have increased, decreased or not changed the number of cigarettes per day smoked during the lockdown.

Concerning e-cigarettes use, students were asked what type of refill they have used (“nicotine”, “aroma”, and “I don't know” were the possible answers). It was a multiple response question and it allows us to identify adolescents who used more than one refill type.

ESPAD#iorestoacasa2020 also investigated the proximity of substance use by asking adolescent “How many of your friends would you estimate…” in regards to the following: “smoke cigarettes or e-cigarettes”, “drink alcoholic beverages”, “get drunk”, “smoke cannabis”, and “other illegal drugs”. The possible answers were “none”, “a few”, “some”, “most”, and “all”. These variables were dichotomized into “no one” = 0 and all the other response options = 1.

Similarly, the questionnaire assessed if parents have used substances through the following question “Do you think one or both of your parents have had some of these experiences?” in relation to the following: “smoke cigarettes or e-cigarettes”, “use drugs without medical prescriptions”, “get drunk”, “smoke cannabis”, and “other illegal drugs”. The possible answers were “no one”, “only father”, “only mother”, “both”, and “I don’t know”. These variables were dichotomized into “at least one parent” = 1 and “no one” = 0.

Risk perception was assessed using the question “How much do you think people risk harming themselves (physically or in other ways), if they …?”, asked in regard to “smoke cigarettes occasionally”, “smoke one or more packs of cigarettes per day”, “have one or two drinks several times in a week”, “have one or two drinks nearly every day”, “get drunk once a week”, and “have five or more drinks in one occasion nearly each weekend”. The possible answers were: “no risk”, “slight risk”, “moderate risk”, “great risk”, “I don’t know”. The responses were dichotomized as “I don’t know” = 0, “no risk” = 1, and “risky” = 3 (slight, moderate, and great risk).

Other variables assessed in the ESPAD#iorestoacasa2020 and ESPAD®Italia 2019 questionnaires were:

1. living area, with the following answer options: “city”, “suburbs”, “village”, and “rural area”;
2. family socioeconomic status assessed by asking “How well-off is your family compared to other families in Italy?”. “very much better off” (1), “much better off” (2), “better off” (3), “about the same” (4), “less well-off” (5), “much less well-off” (6), “very much less well off” (7) were the possible answers, then dichotomized as “low-income family status” = 0 (response options from 5 to 7) or “high-income family status” = 1 (response options from 1 to 4) family socioeconomic status.
3. concerning academic performance, students were asked “Which of the following judgments best describes your school performance in the last quarter?” with the following answers “1 = optimal”, “2 = good”, “3 = medium” and “4 = low”. We dichotomized the answers into “medium–low” (response options from 3 to 4) and “optimal” (response options from 1 to 2).
4. adolescents were asked “In general, how much are you satisfied with yourself?” and the answer options were: “very satisfied”, “satisfied”, “neither satisfied, nor dissatisfied”, “not so satisfied”, “not satisfied at all”. We dichotomized the answers in “being satisfied” = 1 (“very satisfied” or “satisfied”) and “being dissatisfied” = 0 (from “neither satisfied, nor dissatisfied” to “not satisfied at all”).
5. parent–child relationship was asking “How often do the following statements apply to you?” and the following statement were considered “My parents set definite rules about what I can do at home/ outside the home”; “My mother and / or father give me money easily” and “I feel emotionally supported by my mother and/or father”. The answer options were: “almost always”, “often”, “sometimes”, “seldom”, and “almost never”. We dichotomized the answers in “seldom/almost never” = 1 and from “sometimes” to “almost always” = 0.

Students were also asked “How much do you usually spend a week on your personal needs, without parental control?”. The possible answers range from “0€” to “100€ or more” and they were dichotomized into “less than 45€” = 0 and “45€ or more” = 1.

Lastly, adolescents were asked “In general, how satisfied are you with your relationship with your parents?” with the following answers “very satisfied”, “satisfied”, “neither satisfied, nor dissatisfied”, “not so satisfied”, “not satisfied at all”. As well as for the question about self-satisfaction, the answers were dichotomized in “being satisfied” = 1 (“very satisfied” or “satisfied”) and “being dissatisfied” = 0 (from “neither satisfied, nor dissatisfied” to “not satisfied at all”). Remaining within the family context, adolescents were also asked “In general, how much are you satisfied with your relationship with your siblings?” with the same possible answers and dichotomization.

All the variables presented in this section were included in the statistical analysis.

The study complies with the European and national ethics rules and received the ethical approval for its conducting (Research Ethics and Integrity Committee CNR prot. 0027159/2019).

### Statistical Analysis

Propensity Score Matching (PSM) is a quasi-experimental method in which the researcher uses statistical techniques to construct an artificial control group by matching each treated unit with a non-treated unit of similar characteristics (Rosenbaum and Rubin 1983). In our design, students of ESPAD#iorestoacasa 2020 were considered as treated units (cases), whilst students of ESPAD®Italia 2019 were considered as non-treated units (controls). A one-to-one matching was performed using sex, age, macro-area of origin (north-east, north-west, centre and south), living area (city, suburbs, village, and rural area) and family income.

In order to explore factors associated with substance use, univariate analyses controlled for sex and age was performed to test the statistical significance of variables of interest (*p* < 0.05) for both data from 2020 and 2019. Secondly, binary logistic regression models were developed. The use (vs non-use) of each substance were considered as dependent variables and all the variables mentioned in the measure section that resulted to be significantly at univariate analysis were considered independent variables. Finally, sex and age were considered possible confounding variables and were included in each regression. Results are reported as an adjusted odds ratio (OR) with a 95% confidence interval for both years (see Tables 3 and 4).

All analyses were performed using IBM SPSS Statistics Version 26.

## Results

During lockdown, 43.1% of students drank alcohol, 4.2% got intoxicated, and 16.0% had five or more drinks in a row (binge drinking). In 2019 the percentages of monthly alcohol use, intoxication and binge drinking were respectively 63.8%, 12.4%, and 36.8%.

18.4% (33.8% in 2019) of students smoked at least one cigarette during the lockdown period, especially girls (M = 17.1%, F = 19.8%; *p* < 0.05). More in detail: 59.1% smoked at least one cigarette per day and 10.0% smoked more than 10 cigarettes per day. Most of the students (56.0%) reported decreasing their cigarette consumption during the lockdown, while 13.9% increased their consumption and 3.9% changed smoking modality (e.g., starting to smoke e-cigarettes). Moreover, 2.8% (M = 3.2% F = 2.3%; *p* < 0.05) smoked e-cigarettes during lockdown while, in 2019, monthly e-cigarettes consumption was 10.2%.

14.6% of the students used energy drinks and 3.5% used alcohol mixed with energy drinks during the restriction period while the monthly consumptions in 2019 were respectively 34.2% and 16.1%.

7.2% used painkillers (9.2% in 2019). Among them, 14.2% used them to get high and 41.9% used them because they were not satisfied with themselves. 2.9% (4–1% in 2019) of students used medicine without medical prescription during lockdown and 0.8% used them to get high. Regarding illegal substances use, 5.9% used cannabis during lockdown, especially boys (M = 7.2%, F = 4.5%; p < 0.001), while in 2019, 16.0% of the students used cannabis in the last month. Finally, 0.9% of adolescents used at least an illegal substance, with the exception of cannabis (in 2019, 1.6%).

For more detail see Table 2 “Legal or illegal monthly substance consumption in 2019 and 2020”.

**Table 2:** Legal or illegal monthly substance consumption in 2019 and 2020

	2020				2019				Chi-square years
	Male (%)	Female (%)	Total (%)	Chi-square gender	Male (%)	Female (%)	Total (%)	Chi-square gender	
Alcohol use	46.5	39.3	43.1	***	67.0	60.1	63.8	***	***
Alcohol with energy drinks	4.7	2.2	3.5	***	20.3	11.3	16.1	***	***
Intoxication	4.8	3.6	4.2	*	12.4	12.4	12.4	n.s.	***
Binge drinking	16.7	15.3	16.0	n.s.	40.6	32.5	36.8	***	***
Cigarette	17.1	19.8	18.4	*	32.4	35.4	33.8	*	***
Electronic cigarettes	3.2	2.3	2.8	*	12.2	7.9	10.2	***	***
Energy drinks	20.6	8.3	14.6	***	46.0	20.8	34.2	***	***
Drugs without medical prescription	1.9	3.9	2.9	***	2.2	6.2	4.1	***	***
Pain medications	3.7	10.9	7.2	***	5.5	14.7	9.8	***	***
Cannabis	7.2	4.5	5.9	***	19.4	12.2	16.0	***	***
Any other illegal substance	1.4	0.4	0.9	***	2.1	1.0	1.6	**	***

*** *p* < .001; ** *p* < .005; * *p* < .05; n. s. not statistically significant

The use of cigarettes in the last thirty days, for both 2019 and 2020 data, was positively associated with medium–low academic performance, the low satisfaction with relationship with parents, the money spent without parental control, the perception that smoking 10 cigarettes every day is not risky, and perception that friends and/or parents smoked cigarettes or e-cigarettes. In 2019, a positive association was also found concerning the low satisfaction with one's own health (see Table 3a).

**Table 3:** Factors associated with legal substance use

	2019		2020	
	OR (95% CI)	*p* value	OR (95% CI)	*p* value
*a) Use of cigarettes*				
High-income family status	n.s.	n.s.	n.s.	n.s.
Medium–low academic achievement	2.01 (1.74;2.33)	***	2.14 (1.81;2.53)	***
Dissatisfied with relationship with parents	1.40 (1.16;1.70)	***	1.47 (1.20;1.80)	***
Dissatisfied with him/herself	n.s.	n.s.	n.s.	n.s.
Dissatisfied with heath	1.25(1.03;1.53)	*	–	–
Parents lay down some rules on behaviour: sometimes/rarely	n.s.	n.s.	n.s.	n.s.
Parents give money easily: sometimes/rarely	–	–	n.s.	n.s.
Spend more than 45 Euros on individual needs during the week without parental control	3.20 (2.43;4.22)	***	2.98 (2.180;4.08)	***
Think that friends smoke cigarettes or e-cigarettes	3.69 (2.68;5.08)	***	3.30 (2.36;4.63)	***
Perception that it is not risky smoking 10 cigarettes every day	2.33 (1.62;3.34)	***	2.02 (1.27;3.21)	**
Think that parents smoke cigarettes or e-cigarettes	2.21 (1.80;2.73)	***	2.12 (1.68;2.68)	***
*b) Consumption of alcohol*				
Medium–low academic achievement	1.28 (1.11;1.46)	***	n.s.	n.s.
Dissatisfied with relationship with parents	n.s.	n.s.	n.s.	n.s.
Dissatisfied with him/herself	n.s.	n.s.	n.s.	n.s.
Parents lay down some rules on behaviour: sometimes/rarely	1.17 (1.02;1.33)	*	1.33 (1.17;1.51)	***
Parents give money easily: sometimes/rarely	–	–	n.s.	n.s.
Spend more than 45 Euros on individual needs during the week without parental control	2.57 (1.86;3.54)	***	1.80 (1.33;2.44)	***
Think that friends drink alcohol	2.02 (1.51;2.71)	***	1.97 (1.47;2.64)	***
Think that friends get drunk	2.330 (1.890;2.87)	***	1.73 (1.40;2.15)	***
Think my parents get drunk	2.18 (1.88;2.52)	***	1.69 (1.46;1.97)	***
Perception that it is not risky drinking 1 or 2 alcoholic beverages almost every day	1.44 (1.14;1.80)	*	1.54 (1.25;1.90)	***
Perception that it is not risky drinking 5 or more alcoholic beverages consecutively every weekend	1.61 (1.15;2.27)	**	1.98 (1.44;2.71)	***
*c) Intoxication*				
Dissatisfied with relationship with parents	1.36 (1.09;1.71)	**	1.49 (1.02;2.17)	*
Dissatisfied with him/herself	n.s.	n.s.	n.s.	n.s.
Medium–low academic achievement	1.42 (1.17;1.71)	***	1.50 (1.09;2.07)	*
Parents lay down some rules on behaviour: sometimes/rarely	n.s.	n.s.	n.s.	n.s.
Parents give money easily: sometimes/rarely	n.s.	n.s.	n.s.	n.s.
Spend more than 45 Euros on individual needs during the week without parental control	2.44 (1.84;3.25)	***	3.23 (2.05;5.08)	***
Think that friends drink alcohol	n.s.	n.s.	n.s.	n.s.
Think that friends get drunk	7.51 (3.65;15.49)	***	4.21 (1.68;10.55)	**
Think my parents get drunk	2.437 (1.838;3.231)	***	1.58 (1.03;2.42)	*
Perception that it is not risky drinking 1 or 2 alcoholic beverages almost every day	n.s.	n.s.	n.s.	n.s.
Perception that it is not risky drinking 5 or more alcoholic beverages consecutively every weekend	1.82 (1.30;2.53)	***	3.02 (1.82;5.03)	***
*d) Binge drinking*				
Medium–low academic achievement	1.65 (1.46;1.87)	***	1.70 (1.40;2.02)	***
Dissatisfied with relationship with parents	n.s.	n.s.	n.s.	n.s.
Dissatisfied with him/herself	–	–	n.s.	n.s.
Parents lay down some rules on behaviour: sometimes/rarely	n.s.	n.s.	n.s.	n.s.
Parents give money easily: sometimes/rarely	–	–	n.s.	n.s.
Spend more than 45 Euros on individual needs during the week without parental control	2.39 (1.89;3.03)	***	2.29 (1.69;3.11)	***
Think that friends drink alcohol	n.s.	n.s.	n.s.	n.s.
Think that friends get drunk	2.42 (1.89;3.11)	***	2.91 (2.00;4.23)	***
Think my parents get drunk	1.78 (1.53;2.08)	***	1.51 (1.22;1.86)	***
*e) Consumption of alcohol with energy drink*				
Medium–low academic achievement	1.79 (1.51;2.12)	***	n.s.	n.s.
Dissatisfied with relationship with parents	n.s.	n.s.	n.s.	n.s.
Parents lay down some rules on behaviour: sometimes/rarely	1.22 (1.03;1.43)	**	1.67 (1.18;2.36)	**
Spend more than 45 Euros on individual needs during the week without parental control	2.34 (1.80;3.02)	***	3.82 (2.41;6.05)	***
Think that friends drink alcohol	n.s.	n.s.	n.s.	n.s.
Think that friends get drunk	2.55 (1.72;3.78)	***	3.28 (1.36;7.89)	*
Think my parents get drunk	1.83 (1.46;2.28)	***	1.89 (1.17;3.06)	*
Perception that it is not risky drinking 1 or 2 alcoholic beverages almost every day	n.s.	n.s.	n.s.	n.s.
Perception that it is not risky drinking 5 or more alcoholic beverages consecutively every weekend	1.59 (1.17;2.18)	***	2.63 (1.51;4.58)	**
*f) Consumption of drugs without medical prescription*				
High-income family status	n.s.	n.s.	n.s.	n.s.
Dissatisfied with relationship with parents	n.s.	n.s.	n.s.	n.s.
Dissatisfied with him/herself	n.s.	n.s.	n.s.	n.s.
Parents lay down some rules on behaviour: sometimes/rarely	n.s.	n.s.	n.s.	n.s.
Feeling emotionally supported from parents: sometimes/rarely	n.s.	n.s.	1.37 (1.07;1.75)	*
My parents give me money easily: sometimes/rarely	n.s.	n.s.	n.s.	n.s.
Spend more than 45 Euros on individual needs during the week	n.s.	n.s.	2.13 (1.53;2.97)	***
Think that friends use illegal substances	n.s.	n.s.	1.32 (1.08;1.61)	**
Think that parents use drugs without medical prescription	n.s.	n.s.	n.s.	n.s.
Think that parents use drugs with medical prescription	1.837 (1.161;2.907)	*	1.37 (1.04;1.81)	*
Think it is not risky using drugs without medical prescription occasionally	2.48 (1.47;4.18)	*	n.s.	n.s.
Dissatisfied with health	2.25 (1.53;3.29)	***	n.s.	n.s.
*g) Use of painkillers*				
High-income family status	–	–	n.s.	n.s.
Dissatisfied with relationship with parents	n.s.	n.s.	n.s.	n.s.
Dissatisfied with him/herself	n.s.	n.s.	1.32 (1.02;1.71)	*
Dissatisfied with relationship with sisters/brothers	n.s.	n.s.	–	–
Spend more than 45 Euros on individual needs during the week without parental control	–	–	2.10 (1.35;3.26)	**
Think that parents use drugs with medical prescription	1.86 (1.49;2.33)	***	1.47(1.13;1.92)	*
Perception that it is not risky using drugs without medical prescription occasionally	1.94 (1.31;2.86)	**	n.s.	n.s.
Dissatisfied with health	1.33 (1.01;1.74)	*	1.55 (1.16;2.10)	**

***p < .001; ** *p* < .005; * *p* < .05; n. s. not statistically significant

"–" = chi square > .05

Regarding the monthly consumption of alcohol in 2019 and 2020, a positive association was observed with the lack of rules on adolescents’ behaviour, the money spent without parental control, the perception that friends drink alcohol and/or get drunk, thinking that parents get drunk. and a low risk perception related to drinking 1 or 2 alcoholic beverages almost every day and drinking 1 or 2 alcoholic beverages almost every day. Among 2019 samples, positive association emerged between medium–low academic performance and alcohol use (see Table 3b).

To provide a comprehensive and extensive picture of the alcohol drinking patterns, also the number of intoxications, the number of episodes of binge drinking, and the use of alcohol mixed with energy drinks were considered as dependent variables.

For both 2019 and 2020 data, last month intoxication was positively associated with low satisfaction with the relationship with parents, medium–low academic performance, the money spent without parental control, the perception that friends get drunk, thinking that parents get drunk, and a low risk perception related to drinking 5 or more alcoholic beverages consecutively every weekend (see Table 3c).

Logistic regressions related to 2019 and 2020 data showed that binge drinking was positively associated with medium–low academic performance, the money spent without parental control, the perception that friends get drunk, and thinking that parents get drunk (see Table 3d).

In both 2019 and 2020 sample, last month consumption of alcohol mixed with energy drink was positively associated with the lack of rules on adolescents’ behaviour, the money spent without parental control, the perception that friends get drunk, thinking that parents got drunk, and a low risk perception related to drinking 5 or more alcoholic beverages consecutively every weekend. Furthermore, in 2019 the use of alcohol mixed with energy drinks was associated with medium–low academic performance (see Table 3e).

Logistic regression conducted on 2020 data showed that the consumption of non-prescription drugs was negatively associated with feeling emotionally supported by parents and having parents who easily give money to their children. Moreover, it was positively associated with the money spent without parental control and thinking that parents use drugs with medical prescription. In 2019 data, monthly non-prescription drug use was positively associated with thinking that parents use drugs with medical prescription, a low risk perception related to non-prescription drug use, and the low satisfaction with one’s own health (see Table 3f).

In 2020, painkillers use during lockdown was positively associated with low self-satisfaction, the money spent without parental control, thinking that parents use drugs with medical prescription, and the low satisfaction with one’s own health. In 2019, there were associations between monthly painkillers use and thinking that parents use drugs with medical prescription, a low risk perception related to non-prescription drug use, and the low satisfaction with one’s own health (see Table 3g).

In regards to illegal substances, logistic regression conducted on 2019 and 2020 samples showed that monthly cannabis use was positively associated with medium–low academic performance, the low satisfaction with relationship with parents, the lack of rules on adolescents’ behaviour, the money spent without parental control, and perception that friends use illegal substances. In 2019, we also observed a positive association between cannabis use and the low satisfaction with the relationship with siblings, and thinking that parents used illegal substances (see Table 4a).

**Table 4:** Factors associated with illegal substance use

	2019		2020	
	OR (95% CI)	*p* value	OR (95% CI)	*p* value
*a) Use of cannabis*				
High-income family status	n.s.	n.s.	n.s.	n.s.
Medium–low academic achievement	1.94 (1.60;2.35)	***	2.26 (1.68;3.059)	***
Dissatisfied with relationship with parents	1.56 (1.23;1.96)	***	1.810 (1.27;2.58)	**
Dissatisfied with relationship with sisters/brothers	1.32 (1.06;1.66)	*	n.s.	n.s.
Dissatisfied with him/herself	n.s.	n.s.	n.s.	n.s.
Parents lay down some rules on behaviour: sometimes/rarely	1.34 (1.11;1.66)	**	1.41 (1.05;1.89)	*
Parents give money easily: sometimes/rarely	n.s.	n.s.	n.s.	n.s.
Spend more than 45 Euros on individual needs during the week without parental control	2.13 (1.59;2.86)	***	3.93 (2.55;6.06)	***
Think that friends use illegal substances	1.35 (1.11;1.63)	**	1.95 (1.45;2.63)	***
Think that parents use illegal substances	1.59 (1.26;2.02)	***	n.s.	n.s.
*b) Use of any other illegal substances except cannabis*				
High-income family status	n.s.	n.s.	0.33 (0.12;0.94)	*
Dissatisfied with relationship with parents	n.s.	n.s.	n.s.	n.s.
Dissatisfied with relationship with sisters/brothers	n.s.	n.s.	n.s.	n.s.
Spend more than 45 Euros on individual needs during the week without parental control	4.77 (2.34;9.74)	***	7.54 (2.75;20.65)	***
Think that friends use illegal substances	3.58 (1.81;7.08)	***	9.42 (2.67;33.21)	***
Think that parents use illegal substances	3.58 (1.91;6.72)	***	8.54 (3.07;23.77)	***

*** *p* < .001; ** *p* < .005; * *p* < .05; n. s. not statistically significant

"–" = chi square > .05

Finally, in both 2019 and 2020, illegal substance use (cocaine, heroin, hallucinogens, and stimulants) was positively associated with the money spent without parental control, perception that friends use illegal substances, and thinking that parents used illegal substances. Logistic regression conducted on 2020 data, also showed a negative association between illegal substance use and family income (see Table 4b).

## Discussion

This article provides a comprehensive picture of substance use and associated factors during the first COVID-19 lockdown in Italy among high school students (aged 15–19 years).

Our findings show that, compared to the monthly consumption in 2019, during the restriction period a decrease in the consumption of all types of the considered substances (both legal and illegal) is observed. It is possible to argue that COVID-19 restrictions made it more difficult to obtain psychoactive substances, reducing the opportunities for social consumption and the possibility of acquiring substances outside. Despite this, all kinds of psychoactive substances were still used by some students. In particular, the most used was alcohol. This result can be partially explained by the Italian cultural context. Italy belongs to the so-called “wet” cultures (Petrilli et al., 2014; Savic et al., 2016), meaning that it is considered socially acceptable to consume a moderate amount of alcohol with meals (Savic et al., 2016). In accordance with that, during lockdown, parents may find it acceptable for their children to consume alcohol, and they allow it even at home, in their presence and even to the younger ones (aged under 18). However, drunkenness is still condemned by Italian social norms (Petrilli et al., 2014; Savic et al., 2016). Thus, the frequency of binge drinking and intoxication observed in our study could not be linked to Italian culture but it is probably related to the easy availability of alcohol in Italian homes and to the possible feelings of anxiety and discomfort experienced by the youth during lockdown (Blumenthal et al., 2020; Skrzynski & Creswell, 2020).

This study also addresses the factors associated with psychoactive substance consumption, comparing data from 2019 and 2020.

First of all, our results point out that several factors that are considered well known risk factors for substance consumption, still have a significant role during lockdown period (e. g., parental-child relationship, self-esteem, risk perception). This can be considered an interesting result because it underlines that the aforementioned factors are important even during a stressful time, such as a pandemic. Furthermore, they suggest that students with particular frailties are those who continue to engage in risky behaviours (such as substance use) despite the physical and logistical difficulties presented by the restrictions for COVID-19.

More in detail, we did not find any differences in the factors associated with cigarette consumption, intoxication and binge drinking. Concerning cigarette smoking, it may be explained assuming that the habit of smoking or not smoking may be less affected by the restrictions and the students who had started smoking continued to do so, even during the lockdown. In terms of frequency, a higher percentage of teenagers claimed to have decreased the number of cigarettes smoked than those who increased them, despite the period of potential high stress. This phenomenon is probably linked to the greater difficulty in buying cigarettes (Doubeni et al., 2008), the loss of the social component linked to smoking in social context (Ennett et al., 2006; Hoffman et al., 2007; Maxwell, 2002), and the greater parental control (Kiesner et al., 2010) experienced during the restriction period. All these factors are able to reduce tobacco consumption.

This study also points out some changes in factors associated with psychoactive substance use.

In 2019, academic performance was associated with substance consumption and it was confirmed by literature findings (Bryant et al., 2003; Bugbee et al., 2019; Cox et al., 2007; EMCDDA, 2019; Zimmerman & Schmeelk‐Cone, 2003). Nevertheless, in 2020, it loses its significance in the association with the consumption of alcohol and alcohol mixed with energy drinks. During the restriction period, academic achievements may lessen its importance because of distant learning and pandemic issues. Moreover, given the greater accessibility compared with other substances, alcohol mixed with energy drinks may have been used equally by students with high and low academic performance.

During lockdown, a high family income resulted to be negatively associated with illegal substance consumption (i.e., cocaine, heroin, hallucinogens and stimulants), in contrast with what emerged from 2019 data. It is possible to speculate that, during the restrictions, having a more affluent family may have been associated with a better quality of life, in terms of having a larger home and more leisure and entertainment options, through hobbies and electronic devices. In this context, students may have been less prone to feel distress and to engage in risky behaviours, especially those particularly dangerous such as hard drugs consumptions.

Regarding cannabis use, our study shows that, unlike what happened in the similar 2019 sample, during the lockdown students consumed or not cannabis regardless of the relationship with their siblings and regardless of whether they thought that their parents had smoked cannabis or not. Therefore, other factors may have become more relevant, for example stress related to the pandemic situation and restrictions.

Most of the differences in the analyses conducted on the 2019 and 2020 samples emerge with regard to the consumption of non-prescription drugs and painkillers. These types of substances may have distinguished themselves from the others because they may have been found directly in the home. Therefore, they may have been used instead of other psychoactive substances, by students who do not feel emotionally supported by parents or who spend money without parental control and, in the case of painkillers, by students who were dissatisfied with themselves. Moreover, non-prescription drugs and painkillers have been used regardless of the associated risk perception and, in the case of non-prescription drugs, regardless of health and self-satisfaction or dissatisfaction.

All together, these findings suggest that, despite the stressful period, the prevalence of substance use decreases. Nevertheless, restrictions were not able to prevent all substance use, especially among students with frailty. Although there were some changes in the ease of obtaining the substances, students with particular risk factors continued to use them, possibly changing substance type. Focusing on the factors that are associated with psychoactive substance consumption during lockdown or both during and before lockdown, the study may provide some useful direction for preventive campaigns, expanding information for evidence-based intervention and policy (EMCDDA, 2019). They may focus more on social norms and peer influence in order to reduce substance appeal, even during stressful life events. Indeed, group or peer-to-peer interventions may be useful in order to lower the social desirability of substance use and increase risk perception and knowledge. Furthermore, family may play an important role here too (EMCDDA, 2019; Ferri et al., 2013). Considering the forced cohabitations, trying to spend quality time together may be useful to avoid both making adolescents spend time using psychoactive substances or engaging in risky behaviours. Indeed, parents could be a source of emotional support and they may promote better coping strategies and adaptation to the ongoing pandemic.

Given the potential long-term impact that restrictions and the pandemic may have on adolescent well-being (Efuribe et al., 2020; Fegert et al., 2020; Guessoum et al., 2020), the information provided by this research might be relevant in order to better understand adolescents’ substance use and thus provide information for prevention efforts.

### Strengths and Limitations

This study presents some limitations. First of all, we use a self-administered questionnaire with its related problems (e.g. memory recall biases and social desirability biases). Secondly, it is a cross-sectional study so it is not possible to establish causal relationships between analysed variables.

Given that the study is not based on a clinical or users’ population, the prevalence of use of some substances (i. e. stimulants, hallucinogens, opiates and cocaine) is very low. For this reason, logistic regressions were conducted on a small sample. Finally, although large, the sample is not statistically representative of the general Italian high-school student population.

Despite the limitations, this study provides useful information on Italian adolescents’ substance use and associated factors, during a peculiar period like a pandemic and lockdown.
